# Evolution, Complexity, and Life History Theory

**DOI:** 10.1007/s13752-024-00487-z

**Published:** 2025-01-14

**Authors:** Walter Veit, Samuel J. L. Gascoigne, Roberto Salguero-Gómez

**Affiliations:** 1 Department of Philosophy, University of Reading, Reading, UK; 2 Munich Center for Mathematical Philosophy, LMU, Munich, Germany; 3 Department of Biology, University of Oxford, Oxford, UK; 4 School of Biological Sciences, University of Aberdeen, Aberdeen, UK; 5 Centre for Biodiversity and Conservation, University of Queensland, Brisbane, Australia; 6 Evolutionary Demography Laboratory, Max Planck Institute for Demographic Research, Rostock, Germany

**Keywords:** Biological complexity, Entropy, Evolutionary trends, Fitness, Goal directedness, Life history theory, Life history complexity, Optimality, Teleonomy

## Abstract

In this article, we revisit the longstanding debate of whether there is a pattern in the evolution of organisms towards greater complexity, and how this hypothesis could be tested using an interdisciplinary lens. We argue that this debate remains alive today due to the lack of a quantitative measure of complexity that is related to the teleonomic (i.e., goal-directed) nature of living systems. Further, we argue that such a biological measure of complexity can indeed be found in the vast literature produced within life history theory. We propose that an ideal method to quantify this complexity lies within life history strategies (i.e., schedules of survival and reproduction across an organism’s life cycle), as it is precisely these strategies that are under selection to optimize the organism’s fitness. In this context, we set an agenda for future steps: (1) how this complexity can be measured mathematically, and (2) how we can engage in a comparative analysis of this complexity across species to investigate the evolutionary forces driving increases or, for that matter, decreases in teleonomic complexity.

## Introduction

In his seminal 1991 article, Daniel McShea criticized the longstanding conviction among evolutionists, ever since Darwin (1859), that the complexity of species increases over evolutionary time, in addition to the closely related idea of orthogenesis/progressive evolution that there is a goal or directionality to the evolutionary process (see Levit and Olsson 2006). Aiming to question these ideas, McShea argued that there is almost no empirical evidence supporting this belief in a kind of directionality in complexity during evolution and that biologists may simply be misled by their own biased presuppositions. Further, he suggested that research should shift from more theoretical model-building work to empirical inquiries into actual changes in complexity offering several avenues for future research. Unfortunately, such a shift has not yet taken place. Rather, it seems that the interest among evolutionary biologists in the notions of complexity and progress has been waning for at least three decades, with the exception of their own work (McShea and Brandon 2010; McShea 1996a, b, 2021). Despite the skepticism advocated by McShea, however, it appears that biologists (as opposed to philosophers of biology) have nevertheless remained convinced in the consensus idea of an increase in complexity through evolutionary time.

Our core argument in this article is that the natural phenomenon driving these ideas and intuitions regarding the directedness of evolution can be understood in terms of a *teleonomic* (goal-directed) kind of complexity that has been increasing ever since the origin of life. Our emphasis on teleonomic complexity does not mean that other forms of complexity explored by biologists in this debate, such as Maynard Smith and Szathmary (1997) on organismal complexity or the work of Lynch and Conery (2003) on genome complexity, are of no relevance, but that there is a distinct form of complexity related to organisms as goal-directed systems that has not yet been recognized. The goal of this article is to offer a new way of thinking about an increase in biological complexity and the illusion of progress in evolution by viewing them through this lens.

To offer some clarifications, we use Pittendrigh’s (1958) definition of the term “teleonomic,” as an evolutionary replacement of pre-Darwinian teleological explanations, i.e., that life is to be explained in terms of its purpose (often associated with a designer) rather than the mechanisms that gave rise to it. The concepts of goals, purposes, functions, and the like were revolutionized in the light of Darwin’s theory of evolution by natural selection that explained them in causal terms. For instance, the goal of an organism is the maximization of fitness—not because that is true for any living system, but because natural selection has selected for such individuals in the past, which gives us predictive power to theorize about individuals in the present. Thus, as we use “teleonomic” in this article, we define “teleonomic” as the goal-directedness of living systems towards fitness-maximization. While the term teleonomic is also relevant for discussions of the “functions” of traits, that is not the focus of this article, which is also why measures of functional complexity do not successfully capture the goal-directedness of organisms (see McShea 2000 for an overview of this literature). Our goal here is not to subsume all other concepts of complexity under ours, only to highlight that there is a distinctive kind of complexity that is worth investigating in more detail. By using this teleonomic lens, we conceptualize teleonomic complexity in terms of how many paths there are in the strategies that organisms have evolved in order to achieve the goal of fitness maximization (similar to decision-making complexity in economics; see Veit 2023) as well as the relative contribution of each of those paths towards this goal (only paths that contribute to fitness can increase teleonomic complexity).^1^ As anyone can recognize, some of these strategies are more complex than others, and our goal here is to emphasize the need to measure and study this complexity.

Furthermore, such a biological measure of complexity is already available within the rich arsenal of metrics provided by life history theory and comparative demography. In assessing the complexity of life history strategies (broadly conceived), we are provided with a teleonomic measure that assesses the degree of complexity within evolved life history strategies in the pursuit of the goal of fitness-maximization. As we will define biological “progress” here, we will minimally define it in terms of an increase in this life history complexity—without any commitments to more controversial associations with the terms such as the idea of perfection. Our interest is only in trying to give a naturalistically plausible account of why so many biologists were convinced of an increase in complexity, not in trying to vindicate all the excesses of this theorizing. In addition, we conclude by outlining two directions for future research, one concerning how this complexity can be measured mathematically, and the other for how we can engage in a comparative analysis of this complexity across species to gain key insights toward understanding the evolution of organismal complexity.

### Article Outline

This article is structured as follows. In the second section, we outline the debate on the evolution of complexity and argue that we should focus on teleonomic complexity to understand why biologists have remained committed to the idea of an increase in complexity in evolution. In the third section, we discuss how to measure teleonomic complexity: one must turn to life history theory. Finally, section four outlines avenues for further research into the evolution of complexity.

## Complexity and Evolution

We agree with McShea (1991) in that discussions of biological complexity have been present among a long row of evolutionists dating back to Darwin,^2^ Lamarck (1984), Cope (1871), Spencer (1890), Huxley (1953), Rensch (1960), and Simpson (1961), and that these discussions have been of particular importance in the investigation of macroevolutionary trends in paleobiology (Eble 2005; Jablonski 2005; Lowery and Fraass 2019). Despite some critiques of the idea, the last century saw great confidence in the idea that evolution increases complexity, as in this writing by Daniel McShea:

[I]ncreasing complexity is still the conventional wisdom. Clear statements that complexity increases can be found in the work of Stebbins (1969), Denbigh (1975), Papentin (1980), Saunders and Ho (1976; 1981), Wake et al. (1986), Bonner (1988), and others. And lately the new thermodynamic school of thought has added its voice to the chorus: Wicken (1979; 1987), Brooks and Wiley (1988), and Maze and Scagel (1983) have all argued that complexity ought to and does increase in evolution. In my own experience, the consensus extends well beyond evolutionary biology and professional scientists. People seem to know that complexity increases as surely as they know that evolution has occurred. (McShea 1991, p. 303)

Much of the writing on biological complexity has unsurprisingly focused on the evolution and explosion of multicellular life and body plans in the Cambrian. And yet, despite this conventional impression and the search for evidence for this thesis, very little evidence either in favor of or against the hypothesis has been obtained. As McShea (1991) notes, few have actually empirically investigated whether complexity increases with evolutionary time. Yet, there have been many attempts at developing adaptive rationales for why an increase in complexity is beneficial and ought to be expected.

Biologists have long confidently maintained that “organismal” or “biological complexity” will increase throughout evolutionary history. This strange attraction to the idea that complexity inevitably increases with evolutionary time may be especially perplexing since it sits uncomfortably close to older vitalist and teleological views of *progressive evolution* or as it is sometimes called “orthogenesis” (Ruse 2019). It is thus unsurprising that McShea (1996b) has been critical of attempts to revive Herbert Spencer’s ideas of progressive evolution and the adaptive rationales of complexity and mind (Godfrey-Smith 1996), though also noting that the idea of progressive evolution remains “essentially the conventional wisdom even today” (McShea 1996b, p. 469). While we do not agree that the idea of progressive evolution is conventional wisdom today, the seeming increase in complexity in organisms such as during the Cambrian explosion (Valentine et al. 1994) has certainly come to inspire a lot of speculation among biologists about an evolutionary trend towards greater complexity (e.g., Carroll 2001; Zhang et al. 2014). If there is no evidence for an increase in complexity over evolutionary timescales, however, there would appear to be little point in offering an adaptive explanation for a phenomenon that may merely be a myth—perhaps as other critics of the idea alongside McShea (1991), such as Williams (1966), Lewontin (1968), and Hinegardner and Engelberg (1983) hint at, a remainder of earlier hierarchical views of the biological world with humans placed on top that biologists have largely abandoned, though remaining popular among the public and the major transitions literature.

McShea (1991) highlights how both empirical and theoretical studies have lacked rigor. For instance, most studies and perspectives miss concise discussions of what complexity actually means. Admittedly, while the concept has long puzzled philosophers and scientists alike, it seems reasonably clear that complexity is a *phenomenon* in nature. Complexity is, as our colloquial understanding of the term rightly suggests, opposed to the idea of simplicity, but this understanding does not give us much purchase on making the notion precise. Parts of nature can be intuitively placed on a continuum from simplicity to complexity. Most would agree that a frog catching a fly is more complex than a stone washed up at a beach. So one might be hopeful that we could develop a straightforward and unified measure of complexity to capture this phenomena in nature—a way of ranking systems on a single scale of complexity. Yet, attempts to operationalize complexity have resisted consensus despite some interesting developments in measuring the number of part types (e.g., Brinkworth et al. 2023).

We believe that part of the challenge here has been especially due to attempts to provide biologically neutral measures of complexity that could in principle be applied to any nonbiological system. However, while such neutral measures have their dedicated uses, we believe that they miss out on what we think has driven most advocates of the view that natural selection would select for greater complexity. Much of this research has focused on *morphological complexity*, rather than genetic complexity or ecosystem complexity, because it could be measured in fossils. Furthermore, as McShea (1991) points out, the way morphological complexity should be measured has largely been inspired by researchers in information theory whose operationalizations of complexity could be applied to living and nonliving systems alike. However, the complexity that matters for biological systems should be informed by the drivers of evolutionary change, a teleonomic measure of complexity that assesses how the complexity of different strategies organisms have evolved to achieve their goal of fitness maximization. Only in this context does the equivocation of biologists between complexity and progress make sense. This does not mean that other forms of complexity such as morphological complexity are irrelevant or unimportant, but for the question we are interested in addressing, they are merely potential sources of teleonomic complexity; they are not constitutive of it. Under some circumstances, for instance, a more complex morphology may be accompanied by simpler life history strategies. More complex subcomponents of an organism (higher functional complexity) are also likely going to correlate strongly with teleonomic complexity, but there is no necessary connection here. They are conceptually distinct.

In explaining ideas about biological complexity, many have drawn on Shannon’s (1948) information theory published in “A Mathematical Theory of Communication,” sometimes referred to as “Shannon information” or “Shannon entropy.” For instance, Shannon entropy is extensively used to measure species diversity in ecology (Jost 2006). Following Godfrey-Smith (1996), Shannon information can be calculated as follows: for any system that has a finite number of possible states, there is a probability of being in that state *i* denoted as *P*_*i*_, “then the complexity or disorder of the system is measured as: E =  − ∑*P*_*i*_ log_2_ (*P*_*i*_)” (1996, p. 28). If there are few possible states or most of the probability space is exhausted by a few options, entropy or thermodynamic probability is low, i.e., there is little uncertainty. If there are many alternative states with similar likelihoods, however, then uncertainty is high and the system is more complex. The higher the entropy, the higher the (potential) information content of the states. Here, both organisms and environments can be understood as complex or simple using the number and probability of their possible states. However, what these measures are lacking is a link to the “goal” of biological systems, i.e., fitness. While these measures of entropy are certainly useful to capture what we may describe as the uncertainty, variability, changeability, heterogeneity, or disorder of systems (Godfrey-Smith 1996), we are skeptical that it captures the kind of complexity that is important to living systems (Smith 1975). This skepticism is so because, as mentioned above, they do not recognize the complex strategic trade-offs organisms undergo to maximize their fitness. Indeed, in the measure of entropy there is no connection to the biological notion of reproduction and survival, the building blocks of organismal fitness.

Finally, to understand teleonomic complexity, we have to examine the population rather than the individual, a key aspect that is neglected in many such measures of biological complexity. As van Groenendael et al. (1994, p. 2410) note, “Variation in life history traits among individuals within populations is ubiquitous in both plants and animals.” The fitness landscape, which is shaped by underlying demographic rates, is used to quantify our proposed measure of life history complexity. Any inference regarding fitness based on complexity is agnostic of our method. A population can have a high level of complexity and a low fitness and vice versa. What we propose is a tool, specifically a fitness-informed measure of life history complexity, that can be used to ask key questions in life history evolution and population ecology. Nevertheless, the fact that life history strategies can be complex also makes them very difficult to study. As such, we are happy to take up the task to explore other forms of complexity that McShea (1991) has left to the discipline: “I leave it to others to discover the extent to which my remarks apply in other complexity domains” (1991, p. 305). Thus, let us now turn to life history theory.

## Life History Theory and Teleonomic Complexity

Life history theory originated out of the study of the trade-offs between survival and reproduction. Some of these were very simple mathematical models (e.g., Leslie and Lefkovitch matrix population models: Leslie 1945; Lefkovitch 1965), while others were quite complex to understand how the schedules of survival and reproduction can impact fitness (see especially Stearns 1992; Roff 1992). As Veit (2023, p. 13) puts it: “To understand a species’ teleonomic strategy is to understand their species-specific trade-offs between costly investments of resources into development, fecundity, and survival, with fitness providing an ultimate ‘common currency’ for this economic decision problem, or ‘game’ against nature.” Trade-offs are universal and so the so-called Darwinian demon cannot evolve.

Because of the myriad factors that have to be traded off against each other, it is no surprise that Morbeck et al. (1997, p. xi) has nicely described life history theory as providing us with “a means of addressing the integration of many layers of complexity of organisms and their worlds.” It is here that we find ourselves provided with the theoretical means to understand teleonomic complexity. While Lewontin criticized adaptationism for not being able to deal with trade-offs and treating organisms as mere robotic bundles of traits (Lewontin 1985; see also Gould and Lewontin 1979), life-history theory offers an adaptationist framework to make sense of just such trade-offs. These trade-offs can be seen as the result of natural selection shaping traits such that a life history agent is able to pursue their goal of maximizing fitness:

In life-history theory, [...] numerous aspects of an organism’s life-cycle, such as the timing of reproduction or the length of its immature phase, can be understood by treating the organism as if it were an agent trying to maximize its expected number of offspring—or some other appropriate fitness measure—and had devised a strategy for achieving that goal. (Okasha 2018, p. 10)

As evolution gives rise to more complex life history strategies, it is easy to see why many early evolutionists were convinced of the idea of progressive evolution. With fitness-maximization being both the teleonomic “goal” and cause of organisms, life histories allow us to study the varying degrees of complexity organisms use to achieve this goal (e.g., from the relatively simple and fatally semelparous salmon to the relatively complex immortal jellyfish, *Turritopsis dohrnii*, that can reproduce sexually and asexually as well as switch back and forth between sexually mature and sexually immature stages). We, therefore, think that our notion of teleonomic complexity offers an elegant way of explaining the connection between complexity and “progress” that has often been made in this debate without necessarily having to explain it away as a mere cognitive bias or mistake. Importantly, such a teleonomic perspective does not have to imply that increases in complexity are inevitable. Indeed, because increases in complexity are typically associated with costs there is also an evolutionary drive towards simplicity, i.e., organisms developing less complex strategies. Two excellent examples that make this obvious are annualism and dwarfism.

Selection can lead to shifts from lower to higher life history complexity, and vice versa. A significant proportion of animals and plants reproduce over multiple reproductive cycles, but many animals (e.g., beetles, butterflies), and most plant weeds are in fact annual, completing their life cycles in a single breeding season (Hautekèete et al. 2001; Friedman 2020). On the other hand, perenniality corresponds to life cycles that last over a year. A quick question here is whether one should expect natural selection to inevitably move species from annuality to perenniality. This is indeed a question that has kept evolutionary biologists puzzled since the beginning of life history theory (Cole 1954). When the probability of future survival is low (e.g., because predation is high), it makes sense for species to evolve very short life cycles and invest their limited resources in one or a few reproductive events (Reznick et al. 2008). Indeed, empirical evidence exists of some species where evolutionary pressures have “fine tuned” their life history strategies towards lower complexity by shifting from complex trade-offs towards investing everything in a few (or even a single) breeding events (Bena et al. 1998; Fox 1990). Furthermore, species can switch relatively rapidly (in evolutionary terms) from complex to simpler strategies, suggesting that the evolutionary pressures on the costs of more complex life history strategies may be high (Friedman 2020). Similarly, we can find dwarfism in many species, i.e., species becoming significantly smaller through evolutionary time in response to selection. Examples include the pygmy marmoset, *Callithrix pygmaea* (Montgomery and Mundy 2013), which stands in opposition to the common observation that animal size increases over time (Alroy 1998). The selective pressures thought to lead to dwarfism are manyfold, though the most often discussed factor is related to the isolation of breeding populations to islands (Foster 1964). As such, we should not expect some general explanation that can explain changes in life course complexity across all of life, but instead a mix of mechanisms. Our explanations will have to be more fine-grained than those offered by Steiner and Tuljapurkar (2022), who have recently shown that much of the non-environmental and nongenetic variability of phenotypes in a population cannot simply be categorized as neutral in respect to evolution. The variability of life courses within even a single population remains a major evolutionary puzzle (Flatt 2020). Our framework has the potential to help us move closer towards an understanding of how and why life history strategies change over evolutionary time. Thus, let us now turn to how this complexity can be understood in the context of life history theory.

### Life History Strategies and Complexity

A life history strategy is broadly characterized as the collection of fitness-related traits organisms use to persist in their environment. From parental care (Klug and Bonsall 2010) to dispersal (Bonte and Dahirel 2017), a plethora of phenotypes are required to fully characterize life histories across the tree of life. Simply put, a life history strategy is not a physical characteristic of a population that one can extract and manipulate. In turn, when we discuss a life history strategy we must require our discourse to be general across temporal and spatial scales. Life histories are combinations of life history traits (Capdevila and Salguero-Gómez 2021), and the latter refer to key moments along the life cycle of a species (e.g., age at maturity, frequency of reproduction, rate of development and generation time; Stearns 1992).

**Fig. 1:**
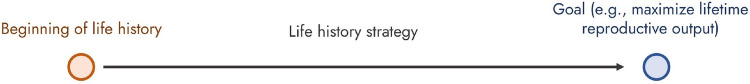
The goal of life history strategies

With this in mind, we propose to define a life history strategy as the time points and actions across an individual’s lifespan that together, across all individuals in a population, allow the population to persist in the face of ecological disturbances. Using this definition, in Fig. 1 we build the archetype of a life history strategy—in its simplest form.

All life history strategies are defined by a schedule starting from the start of a life history (e.g., birth, fission, cloning) and ending in the inevitable: death. The interim, between birth and death, is characterized by a life history strategy that directs the individual towards a simple goal: maximizing lifetime reproductive output or inclusive fitness.

**Fig. 2:**
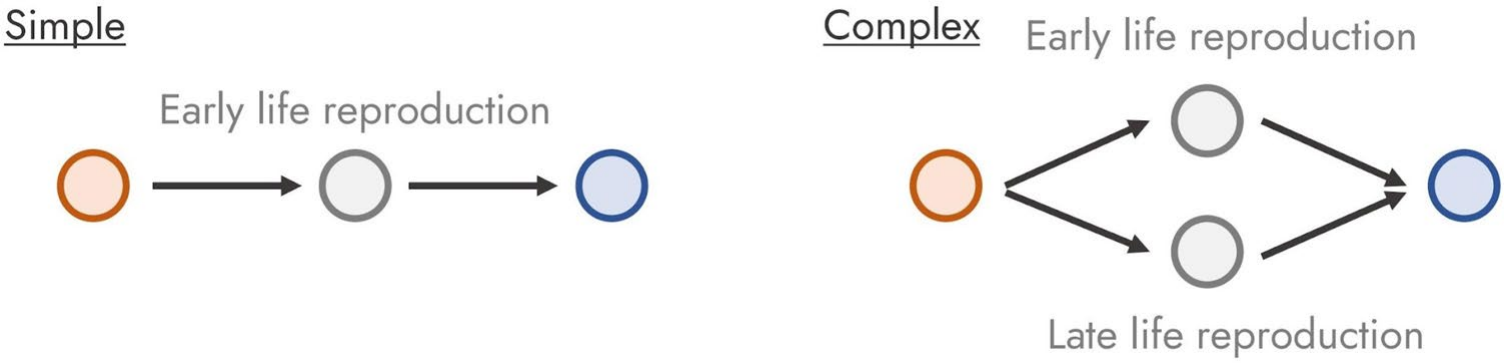
Complex and simple life history strategies

Now that we have built our archetypal life history strategy, let us explore life history complexity. We can define life history complexity as being informed by two components of the aforementioned life history strategy. Firstly, life history complexity is informed by the number of paths individuals of the same population can take from the beginning of their life history to their goal—a term known as individual heterogeneity in life history theory (Tuljapurkar et al. 2009; Vindenes and Langangen 2015). Secondly, life history complexity is informed by the relative contribution of each of the paths toward the goal. For example, Fig. 2 shows two life history strategies with different levels of life history complexity due to the number of possible paths.

**Fig. 3:**
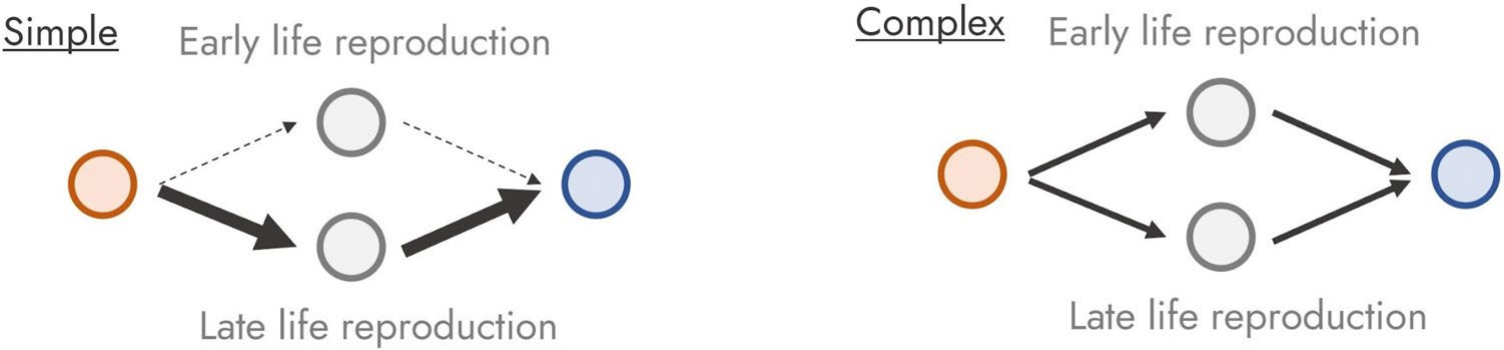
Complex and simple life history strategies

Furthermore, Fig. 3 shows two life history strategies that differ in their complexity based on the evenness in importance of paths for individuals to reach their goal.

In short, by analyzing the number and importance of paths in a life history, we can (even if only relatively) create a framework for life history complexity that is both based on the necessary properties of a life history strategy—shown in the archetypal example—and scalable across modes of life history research (e.g., from demography to behavioral ecology to developmental biology).

### Matrix Population Models and Loop Elasticities

The above framework is especially useful as it allows for a quantitative, rather than qualitative, study of life history complexity across species, populations, and environmental conditions. Specifically, we argue that matrix population models—a discrete time stage/age structured mathematical model where the demographic rates of survival, growth, and reproduction are coerced into matrix form (Caswell 2001)—represents the ideal tool for the mathematical quantification of life history complexity across taxa. As van Groenendael et al. (1994) argued, matrix population models are useful for the analysis of complex life history strategies (see also van Groenendael et al. 1988), which is why we propose them as an ideal resource to measure life history complexity.

Within the many tools that currently exist for the interpretation of matrix population models, elasticities are of particular interest here. Elasticities measure the impact that a relatively small change in a demographic process (e.g., growth) of a given stage represented in the life cycle of a species would cause to the overall population growth rate (de Kroon et al. 1986, 2000). Elasticities have been used to characterize the key elements in the life cycle that influence the overall fitness of a population the most. As such, these approaches have had numerous applications in ecology (Franco and Silvertown 2004), evolution (van Tienderen 2000), and conservation biology (Baxter et al. 2006). However, elasticities only measure “isolated” changes in the demography of a population. In contrast, loop elasticities (van Groenendael et al. 1994) represent the impact on fitness of changing by a small relative amount demographic processes along the life cycle that together constitute a strategy (Fig. 2). As such, we argue that loop elasticities represent the ideal tool for quantifying life history complexity (Fig. 3). The utility of loop elasticities for life history complexity research arises from its mathematical properties. Loop elasticities (${L}_{i}$) are defined as a set indexed by i from 1 to *n*, where *n* represents the total number of life history strategies that can be realized by an organism, given its life cycle. For example, Figs. 2 and 3 represent life histories where *n* = 2. Furthermore, the set of loop elasticities for a given organism sum to 1, thus making them measures of relative importance. To quantify life history complexity, we argue for the combination of loop elasticities and Shannon entropy. We propose a new measure of life history complexity—termed loop entropy (${H}_{L}$)—that is calculated by taking the normalized Shannon entropy of the set of loop elasticities: ${H}_{L}=-\frac{\sum {L}_{i} log ({L}_{i})}{n}$.

This metric has the potential to contextualize life history complexity in eco-evolutionary studies. For example, the *H*_*L*_ measure of life history complexity can be calculated for any species for which stage-structured demographic information exists (e.g., a matrix population model (Caswell 2001), life tables (Pearl and Reed 1920), and integral projection models (Easterling et al. 2000)). In turn, between-species comparisons of life history complexity, as well as phylogenetic approaches (Freckleton et al. 2011), will be key to demonstrate the role of evolutionary pressures in generating more or less complex life history strategies over evolutionary time (e.g., Zanne et al. 2014; Clark et al. 2023). A second future line of research comes in the form of potential versus realized life history complexity within a species. Just as physiological and behavioral traits can be phenotypically plastic (Snell-Rood 2013; Gascoigne et al. 2022; Vinton et al. 2022, 2023), so can the degree of life history complexity realized in a population. Therefore, our *H*_*L*_ measure of life history complexity may vary as a function of local abiotic (e.g., temperature, precipitation) and biotic (e.g., intraspecific competition, predation/prey availability), as substantial intraspecific variation in demographic rates has been found previously (Gaillard et al. 1998; Jongejans et al. 2010). Both the between-species and within-species lines of research represent exciting avenues to further our understanding of life history complexity with the application of loop elasticities.

## Conclusion and Further Directions

Our goal in this article was to introduce a set of conceptual ideas on how to assess a distinctive kind of biological complexity unique to living systems that we have called *teleonomic complexity*. In much of the literature, there has been an unfortunate presupposition that we should think of ideas about the evolution of complexity as being about morphological complexity. Yet, we have argued that the seemingly progressive evolution views of these authors can be naturalized in a less problematic sense in terms of an increase in teleonomic complexity without thereby invoking the idea of orthogenesis. As we hope to have made clear here, the apparent belief of many evolutionists in progress towards greater complexity can in principle be naturalized in a minimalist Darwinian way by restating this thesis as one about an increase in teleonomic complexity. That is, over evolutionary time, more complex life history strategies will emerge, which isn’t to deny strategies often become simpler.

That this complexity should be measured through the lens of life history theory was the second argument of our article. All species have evolved life history strategies to achieve their teleonomic goals of maximizing their genetic representation in the next generation. These fitness differences can be mapped out in different ways to assess the diversity of life and one important dimension along which we can assess this diversity is of course complexity. Some life history strategies are more complex than others and natural selection is leading to an ever-growing exploration of more complex life history strategies (Giménez et al. 2004; Sebert-Cuvillier et al. 2007; Higgins et al. 2015). We are, of course, not endorsing the simplistic orthogenesis view that evolution leads to perfection and greater complexity as an end in itself. However, complex design solutions to the problems animals, plants, and other organisms face do not come out of nowhere. Natural selection provides an entirely unproblematic kind of “progress” if it is defined in a teleonomic manner, since we can expect it to come up with new and more “ingenious” strategies that make sense of the apparent directedness of evolution. We have thus argued against the suggestion by McShea that biologists may have fallen victim to their own cultural and perceptual biases forcing *scala naturae* thinking into our view of life. Progress may be an illusion, but this illusion is driven by real increases in teleonomic complexity.

While McShea depicts theoretical work dismissively, he was certainly right that there is a need for more empirical work to fill out what has largely remained a data and inference vacuum. We are cautiously optimistic that teleonomic complexity can be expected to increase over evolutionary time, yet we acknowledge the need to provide further evidence for this view both in virtue of theoretical models and empirical studies. Future works should apply our new life history complexity measure, the loop entropy, to existing, large arcs of structured demographic information, like the COMADRE (Salguero‐Gómez et al. 2016a) and COMPADRE (Salguero‐Gómez et al. 2015) databases. However, this ability for comparative analysis is not just limited to evolutionary history. Since our proposed measure of life history complexity is agnostic as to where the source of variation arises (unlike morphological metrics like body size (Smith et al. 2016), or brain size (Burger et al. 2019)), future work can analyze contributions from other known sources of life history variation (e.g., genetic heterogeneity and phenotypic plasticity). In particular, further research can relate our proposed measure of life history complexity to the two primary axes of life history variation across plants and animals (i.e., fast-slow continuum and reproductive schedule) using phylogenetically corrected principal component analyses (as in Salguero‐Gómez et al. 2016b).

Finally, we hope that our article will raise interest in the teleonomic complexity of different species, which should not be confused with other notions such as morphological or functional complexity. Indeed, it may help us to explore the connections between these different kinds of complexity to the goal-directedness of organisms. It is our hope that both biologists and philosophers will contribute to its investigations in order to understand under which conditions life history strategies become more complex or, for that matter, more simple.

## Data Availability

A data availability statement is not applicable for this article as not data nor code were used.

## Footnotes

1 One of our goals of this project and collaboration is to offer a mathematical measure for what one of us has dubbed “pathological complexity” in this journal (Veit 2022a) and a monograph (Veit 2023) as a general life-history complexity measure for the purposes of a science of animal consciousness. However, since this article aims to show that biologists should have a general interest in this measure independent of its intended purpose in animal minds research, we chose not to develop these ideas further here to allow the arguments of this article to stand on their own (though see Veit 2022b).

2 Though as McShea (1991) notes, Darwin only discussed his views on macroevolutionary trajectories toward complexity in his Notebook E, not in his *Origin* (see Darwin 1987, p. 422).
